# Associations among Neurofilament light chain (NfL), Depressive Symptoms and Cognition in autosomal dominant Alzheimer´s disease

**DOI:** 10.1002/alz.093350

**Published:** 2025-01-09

**Authors:** David Fernando Aguillon, Daniel Vasquez, Ana Y Baena, Clara Vila‐Castelar, Natalia Acosta‐Baena, Gloria Garcia, Margarita M. Giraldo‐Chica, Victoria Tirado, Claudia Muñoz, Claudia F Guzman, Jairo E. Martinez, Averi Giudicessi, Stephanie Langella, Francisco Lopera, Jennifer R. Gatchel, Yakeel T. Quiroz

**Affiliations:** ^1^ Grupo de Neurociencias de Antioquia, Universidad de Antioquia, Medellín Colombia; ^2^ Grupo de Neurociencias de Antioquia, Facultad de Medicina, Universidad de Antioquia, Medellín, Antioquia Colombia; ^3^ Massachusetts General Hospital, Harvard Medical School, Boston, MA USA; ^4^ Grupo de Neurociencias de Antioquia, Facultad de Medicina, Universidad de Antioquia, Medellín Colombia

## Abstract

**Background:**

Depressive symptoms are associated with neurodegeneration in individuals at increased risk for late‐onset Alzheimer’s disease, but these relationships are less clear in younger individuals and those at risk for autosomal dominant Alzheimer’s Disease (ADAD). Neurofilament light chain (NfL) is a biomarker of axonal damage and neuronal degeneration that becomes abnormal several decades before clinical onset in ADAD. To address this gap, we investigated the associations among depressive symptoms, plasma NfL and cognition in a younger cohort affected by the *PSEN1*‐E280A genetic variant. Carriers of this variant usually develop dementia by their 50s.

**Method:**

A total of 677 carriers (574 cognitively unimpaired and 103 cognitively impaired) and 620 non‐carriers (median age 32 [Interquartile range IQR 24‐42], 55% women, 8 median education years [IQR 4‐11] from the Colombian kindred with ADAD due to the *PSEN1*‐E280A mutation were included. Participants underwent a neuropsychological evaluation, which included the Mini‐Mental State Examination (MMSE), a Spanish version of the Consortium to Establish a Registry for Alzheimer’s Disease (CERAD) word list learning test (WLL), the 15‐item Geriatric Depression Scale (GDS), and the Functional Assessment Staging Test (FAST) to assess functional independence. Mann‐Whitney‐Wilcoxon test was used to examine group differences, and Spearman’s correlations were used to investigate associations among depressive symptoms, NfL and cognition.

**Result:**

Compared to non‐carriers, carriers had higher levels of NfL (carriers=10.98, IQR 5.34‐13.44; non‐carriers=7.41, IQR 4.68‐8.90, p<0.001). There were no group differences in depressive symptoms (carriers =3.44, IQR 1‐5; non‐carriers=2.73, IQR 1‐4, p=0.19). In carriers, higher NfL levels were associated with higher GDS scores (r=0.093; p<0.001). Higher GDS scores were associated with lower MMSE scores (r= ‐0.18; p<0.001) and poorer CERAD WLL performance (r=‐0.17; p<0.001).

**Conclusion:**

Our study showed that higher NfL plasma levels are associated with higher levels of self‐reported depressive symptoms in individuals with ADAD. These findings support neurodegenerative pathology underlying neuropsychiatric symptoms in ADAD and that these relationships can be detected using plasma measures. Future longitudinal analyses, examining other pathological mechanisms, and GDS‐items are needed to understand these symptoms more clearly in individuals with ADAD and whether they may be useful targets for AD prevention efforts.

